# A Meta-Analysis of Serological Response Associated with Yellow Fever Vaccination

**DOI:** 10.4269/ajtmh.16-0401

**Published:** 2016-12-07

**Authors:** Kévin Jean, Christl A. Donnelly, Neil M. Ferguson, Tini Garske

**Affiliations:** 1 MRC Centre for Outbreak Analysis and Modelling, Department of Infectious Disease Epidemiology, Imperial College London, London, United Kingdom

## Abstract

Despite previous evidence of high level of efficacy, no synthetic metric of yellow fever (YF) vaccine efficacy is currently available. Based on the studies identified in a recent systematic review, we conducted a random-effects meta-analysis of the serological response associated with YF vaccination. Eleven studies conducted between 1965 and 2011 representing 4,868 individual observations were included in the meta-analysis. The pooled estimate of serological response was 97.5% (95% confidence interval [CI] = 82.9–99.7%). There was evidence of between-study heterogeneity (*I*^2^ = 89.1%), but this heterogeneity did not appear to be related to study size, study design, or seroconversion measurement or definition. Pooled estimates were significantly higher (*P* < 0.0001) among studies conducted in nonendemic settings (98.9%, 95% CI = 98.2–99.4%) than among those conducted in endemic settings (94.2%, 95% CI = 83.8–98.1%). These results provide background information against which to evaluate the efficacy of fractional doses of YF vaccine that may be used in outbreak situations.

## Introduction

Yellow fever (YF) is a mosquito-borne viral hemorrhagic fever with a high case-fatality ratio. Around 90% of the global burden occurs in Africa, where the disease causes an estimated 80,000 deaths annually.^1^ The ongoing outbreak in Angola with 3,552 suspected and 875 confirmed cases between December 2015 and July 2016 demonstrates the potential for major epidemics and raises fears over global spread to previously unaffected regions.^2^ Although no specific treatment exists, a safe and efficacious vaccine is available, which was developed in the 1930s and has been widely used since.^3^

YF vaccination is recommended for persons ≥ 9 months of age, living in or traveling to high-risk areas. Based on a recent literature review, the World Health Organization (WHO) stated that a single dose of the vaccine is highly immunogenic and confers life-long protection against YF.^4^,^5^

The YF vaccine is considered to be highly efficacious, but currently no pooled efficacy estimate exists. YF burden estimates and projections need to account for past and future vaccination coverage. In the absence of efficacy estimates, these burden estimates usually rely on the assumption of total protection after vaccination, with sensitivity analyses of limited scope.^1^ Integrating a pooled estimate with uncertainty around vaccine efficacy would help better inform strategic use of the vaccine. In the current situation of global vaccine shortage, in the face of a major outbreak, the use of fractional dosing has been approved by WHO in principle; however, the evaluation of the short- and long-term efficacy of fractional dosing will benefit from a solid understanding of the efficacy of the full dose. Based on a recently published systematic literature review,^4^ we present a meta-analysis of serological response rate associated with the YF vaccine.

## Materials and Methods

### Study selection.

Gotuzzo and others recently published a systematic literature review that informed the 2013 WHO position paper on the use of YF vaccine.^4^,^5^ In this paper, we considered the same 12 studies conducted between 1965 and 2011 that were published in 11 articles.^6^–^16^

As assessed by Gotuzzo and others, no studies were excluded from the meta-analysis based on study design criteria, type of correlate of protection or assay used to measure serological response, study quality, or risk of bias. However, Gotuzzo and others identified one study that presented a very low serological response rate. As this low level of response may be linked to operational failure during the evaluated vaccination campaigns, we excluded it from the meta-analysis.^7^

Abstract and full texts of the studies were independently read by two of the coauthors to classify studies according to study population, seroconversion endpoint, study setting (endemic or nonendemic), and study design (interventional, i.e., vaccine was administered within the study framework, or observational, i.e., participants were classified based on their reported vaccination status).

### Outcome measurement.

All studies evaluated vaccine efficacy in humans indirectly as the proportion of vaccinees that seroconverted using different assays to measure neutralizing antibodies (Table 1). Two studies used plaque reduction neutralization tests (PRNTs) with a cutoff for seropositivity defined as log neutralization index (LNI) ≥ 0.7.^9^,^13^ This cutoff was previously reported by protection studies in nonhuman primates as the antibody titer required to protect against lethal challenge.^17^ Four studies used positive PRNT test with antibody titer ≥ 1:10 as seroconversion cutoff.^8^,^10^,^14^,^16^ This titer is generally considered to be associated with protective immunity.^4^ The remaining studies reported seroconversion endpoints less clearly linked with protection.

### Data analysis.

We used the R package metaphor for analysis.^18^ Between-study heterogeneity was assessed by the Cochran's Q test and *I*^2^ statistic. We combined the results using a random effects meta-analysis. Sensitivity analyses were conducted to assess the stability of the pooled estimate to inclusion of individual studies as well as the effect of study size and studied populations. Asymmetry in the funnel plot was examined visually and tested using Egger's test.^19^

Additionally, we conducted a subgroup analyze based on studies using a well-defined seroconversion cutoff consensually considered to confer protective immunity.^8^–^10^,^13^,^14^,^16^ We also stratified individual studies by study design (interventional versus observational) and by study setting (endemic versus nonendemic). We used meta-regression to test for subgroup differences in serological response rates.

## Results

**Figure 1.**
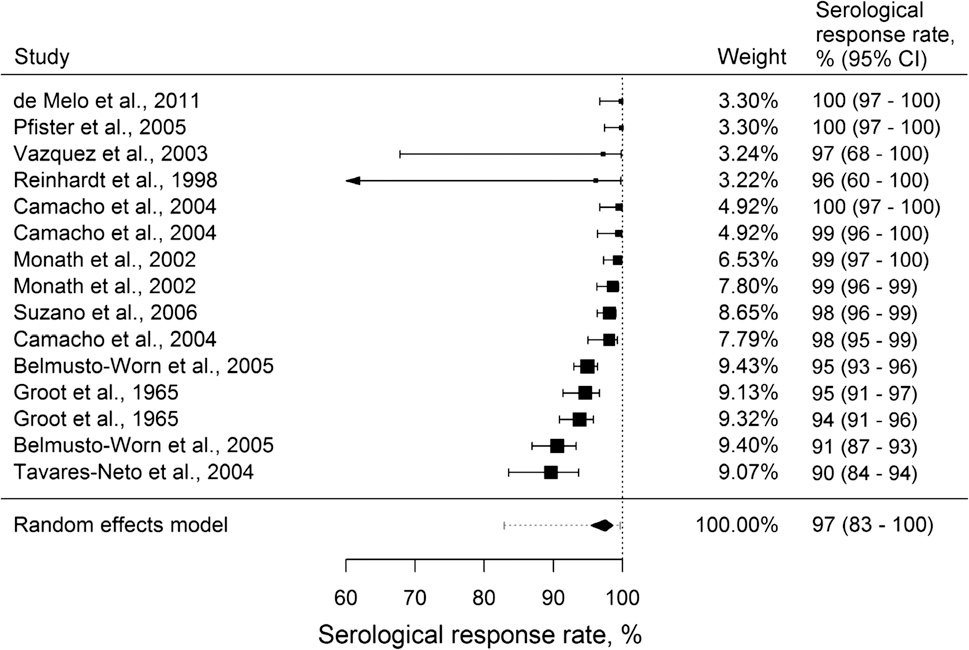
Forest plot of serological response rates after yellow fever vaccination. The diamond delimits the 95% confidence interval (95% CI) of a fixed effects model. Random effects pooled estimate: 97.5% (95% CI = 82.9–99.7%).

The 12 studies analyzed reported serological response rates after vaccination among 15 different treatment groups, representing a total of 4,868 individual observations (Table 1). Across these groups, point estimates ranged from 90 to 100% (Figure 1

).

There was evidence of heterogeneity in serological response between studies (Q test *P* < 0.001; *I*^2^ = 89.1%). The random effects meta-analysis estimated a pooled efficacy of 97.5% (95% confidence interval [CI] = 82.9–99.7%).

The sensitivity analysis confirmed the stability of the pooled estimate, which ranged from 97.2% to 97.8% when excluding individual studies. Sample size did not influence the pooled estimate strongly. When restricting the analysis to studies with > 150 or > 300 participants, the pooled estimates were 97.9% (95% CI = 84.8–99.7%) and 97.7% (95% CI = 84.8–99.7%), respectively. When restricting the analysis to studies conducted in healthy adults, we obtained a pooled estimate of 98.4% (95% CI = 89.1–99.8%).

Visual inspection of the funnel plot (Supplemental Figure 1) and Egger's test presented evidence of asymmetry (*P* < 0.0001).

### Subgroup analyses.

Significant heterogeneity remained when restricting to studies with a seroconversion cutoff consensually considered to confer protective immunity (Q test *P* ≤ 0.001; *I*^2^ = 89.1%; pooled estimate: 98.1%, 95% CI = 79.9–99.8%). Subgroup analysis based on the study design criteria yielded similar results, with evidence of heterogeneity in both observational and interventional studies (for both groups: Q test *P* ≤ 0.001 and *I*^2^ > 80%). Pooled estimates were not significantly different between observational and interventional studies (*P* = 0.283).

Restricting the analysis to studies conducted in endemic settings or settings at transitional risk gave similar results to the main analysis (Q test *P* < 0.001; *I*^2^ = 84.2%; pooled estimate: 94.2%, 95% CI = 83.8–98.1%). However, studies conducted in nonendemic settings exhibited a higher pooled estimate (98.9%, 95% CI = 98.2–99.4%, *P* < 0.0001) with no evidence of heterogeneity (Q test *P* = 0.467; *I*^2^ = 0%).

## Discussion

Based on studies representing 4,868 individual observations, we estimated a pooled serological response rate after vaccination of 97.5%, with 95% CI = 82.9–99.7%. Results were similar when restricting the analysis to studies with a seropositivity cutoff consensually considered as associated with protective immunity. Thus, this pooled estimate may be a good estimate for high protective efficacy of the YF vaccine and is consistent with a previous literature review and with the up-to-date WHO position,^4^,^5^ while carrying a considerable uncertainty which is mostly driven by between-study heterogeneity.

All studies considered here yielded serological response rates of 90% or more. Nonetheless, significant between-study heterogeneity existed, which largely accounts for the uncertainty surrounding the pooled response rate. The source of such heterogeneity is not obvious. Neither differences in study size, design, or population, nor the chosen endpoint for seropositivity satisfactorily explain the heterogeneity in the results. Study setting was the only parameter explaining between-study heterogeneity, with studies conducted in nonendemic setting exhibiting less heterogeneity than studies conducted in endemic setting or settings at transitional risk.

Lower response rate in endemic settings could be partly explained by a differential selection bias. In some of the studies conducted in endemic or low-risk settings, participants with preexisting immunity against YF were excluded from the analysis.^11^,^13^ Thus, participants that were previously exposed to YF but who did not have preexisting immunity, due to a weaker immune system, for example, may be slightly more likely to have been included in these studies. Heterogeneity in the results of studies conducted in endemic settings may thus be linked to heterogeneity in the overall exposure to YF. In contrast, such a selection bias is unlikely in nonendemic settings as previous exposure to YF may be exceptional. This interpretation would imply that heterogeneity observed in the overall meta-analysis was due to study constraints rather than the vaccine itself.

We observed some evidence of publication bias associated with our results. However, sensitivity analysis based on exclusion of individual studies or based on sample size did not show a high dependence of our results on any particular study or study size. We thus think that publication bias is unlikely to have distorted our results.

The pooled estimate relied on studies that were mostly conducted among healthy adults. Previous evidence suggested weaker immune response in specific groups, such as human immunodeficiency virus–infected people or infants.^4^ Specifically, vaccine efficacy in infants and children when coadministered with vaccination against measles, mumps, and rubella, has been recently questioned.^20^ These questions deserve further research effort.

More than 250 million doses of the YF vaccine have been administered in Africa since the 1940s.^1^ However, no previous study has synthetized the evidence to quantify the efficacy based on all available data. In the context of limited resources which holds for most of the endemic zone for YF, a summarizing metric of vaccine efficacy, and maybe more importantly, a measure of the associated uncertainty, is highly welcome. This may be further integrated into vaccine impact evaluation methods and ultimately into the decision process of health resource allocation. Furthermore, it also provides background information against which to evaluate ongoing investigations of the efficacy of a fractional dose approach that may be used in outbreak situations to combat global vaccine shortages.^21^

## Supplementary Material

Supplemental Figure.

## Figures and Tables

**Table 1:** Studies included in the meta-analysis

Study	Publication year	Study design	Study setting	Country	Vaccine assessed	Manufacturer or product	Vaccine potency	Serological assay used	Cutoff used to define seroconversion	Differential test against other flavivirus	Responders /Total sample size	Response rate, % (95% CI)
Groot and Galvis^6^	1965	Observational	Endemic	Colombia	17D†	French neurotropic virus	Not available	NT test in mice	Not available	Not available	282/298	94.6 (91.5–96.7)
Groot and Galvis^6^	1965	Observational	Endemic	Colombia	17D‡	French neurotropic virus	Not available	NT test in mice	Not available	Not available	363/387	93.8 (90.9–95.8)
Guerra and others*^7^	1997	Observational	Endemic	Brazil	17D	Oswaldo Cruz Institute	Not available	NT test in mice	Not available	Not available	131/173	75.7 (68.8–81.5)
Reinhardt and others^8^	1998	Interventional	Nonendemic	Germany	17D	Robert Koch-Institute	4.7 log_10_ PFU	PRNT	NT titer ≥ 1:10	Dengue type 1	12/12	100 (75.8–100)
Monath and others^9^	2002	Interventional	Nonendemic	United States	17D	ARILVAX (PowderJect Pharmaceuticals, United Kingdom)	4.4 log_10_ PFU	PRNT	LNI ≥ 0.7	St. Louis encephalitis, dengue-2, Ilheus, and West Nile	279/283	98.6 (96.4–99.4)
					17D	YF-VAX (Sanofi Pasteur, Swiftwater, PA)	5.0 log_10_ PFU	PRNT	LNI ≥ 0.7	St. Louis encephalitis, dengue-2, Ilheus, and West Nile	289/291	99.3 (97.5–99.8)
Vazquez and others^10^	2003	Interventional	Nonendemic	Cuba	17D	Not available	Not available	PRNT	NT titer ≥ 1:10	Dengue (4 serotypes)	17/17	100 (81.6–100)
Tavares-Neto and others^11^	2004	Observational	Endemic	Brazil	17D	Biomanguinhos	Not available	HI antibodies	Not available	Dengue (4 serotypes), Saint Louis, Ilheus, Rocio	130/145	89.7 (83.6–93.6)
Camacho and others^12^	2004	Interventional	Nonendemic	Brazil	17DD§	Oswaldo Cruz Institute	≥ 1,000 MLD_50_	PRNT	NT titer ≥ 630 mIU/ml	none	205/209	98.1 (95.2–99.3)
					17DD§	Oswaldo Cruz Institute	≥ 1,000 MLD_50_	PRNT	NT titer ≥ 630 mIU/ml	none	192/193	99.5 (97.1–100)
					17D	Oswaldo Cruz Institute	≥ 1,000 MLD_50_	PRNT	NT titer ≥ 630 mIU/ml	none	210/211	99.5 (97.4–100)
Belmusto-Worn and others^13^	2005	Interventional	Transitional risk	Peru	17D	ARILVAX (PowderJect Pharmaceuticals, United Kingdom)	4.4 log_10_ PFU	PRNT	LNI ≥ 0.7	Dengue (4 serotypes)	619/652	94.9 (93.0–96.4)
					17D	YF-VAX (Sanofi Pasteur, Swiftwater, PA)	5.0 log_10_ PFU	PRNT	LNI ≥ 0.7	Dengue (4 serotypes)	298/329	90.6 (86.9–93.3)
Pfister and others^14^	2005	Interventional	Nonendemic	Switzerland	17D	Three different manufacturers	4.2 log_10_ PFU	PRNT	NT titer ≥ 1:10	none	304/304	100 (98.8–100)
Suzano and others^15^	2006	Observational	Endemic	Brazil	17DD	Oswaldo Cruz Institute	Not available	PRNT	NT titer ≥ 630 mIU/ml	none	425/433	98.2 (96.4–99.1)
de Melo and others^16^	2011	Observational	Nonendemic	Brazil	17DD	Oswaldo Cruz Institute	6.3 log_10_ PFU	PRNT	NT titer ≥ 1:20	Dengue (serotype not available)	238/238	100 (98.4–100)

CI = confidence interval; HI = hemagglutination inhibition; LNI = log neutralization index; MLD = minimal lethal dose; NT = neutralization test; PFU = plaque-forming unit; PRNT = plaque reduction neutralization test.

* Not included in the meta-analysis.

† Subcutaneously or by scarification.

‡ By scarification.

§ Different seed lots.
