# Supplementary material for: A Meta-Analysis of Serological Response Associated with Yellow Fever Vaccination
Source: Am J Trop Med Hyg. 2016 Dec 7;95(6):1435–9. doi: 10.4269/ajtmh.16-0401 (PMC5154464; doi:10.4269/ajtmh.16-0401)
Supplement: Supplementary file 1 [file SD8.pdf]

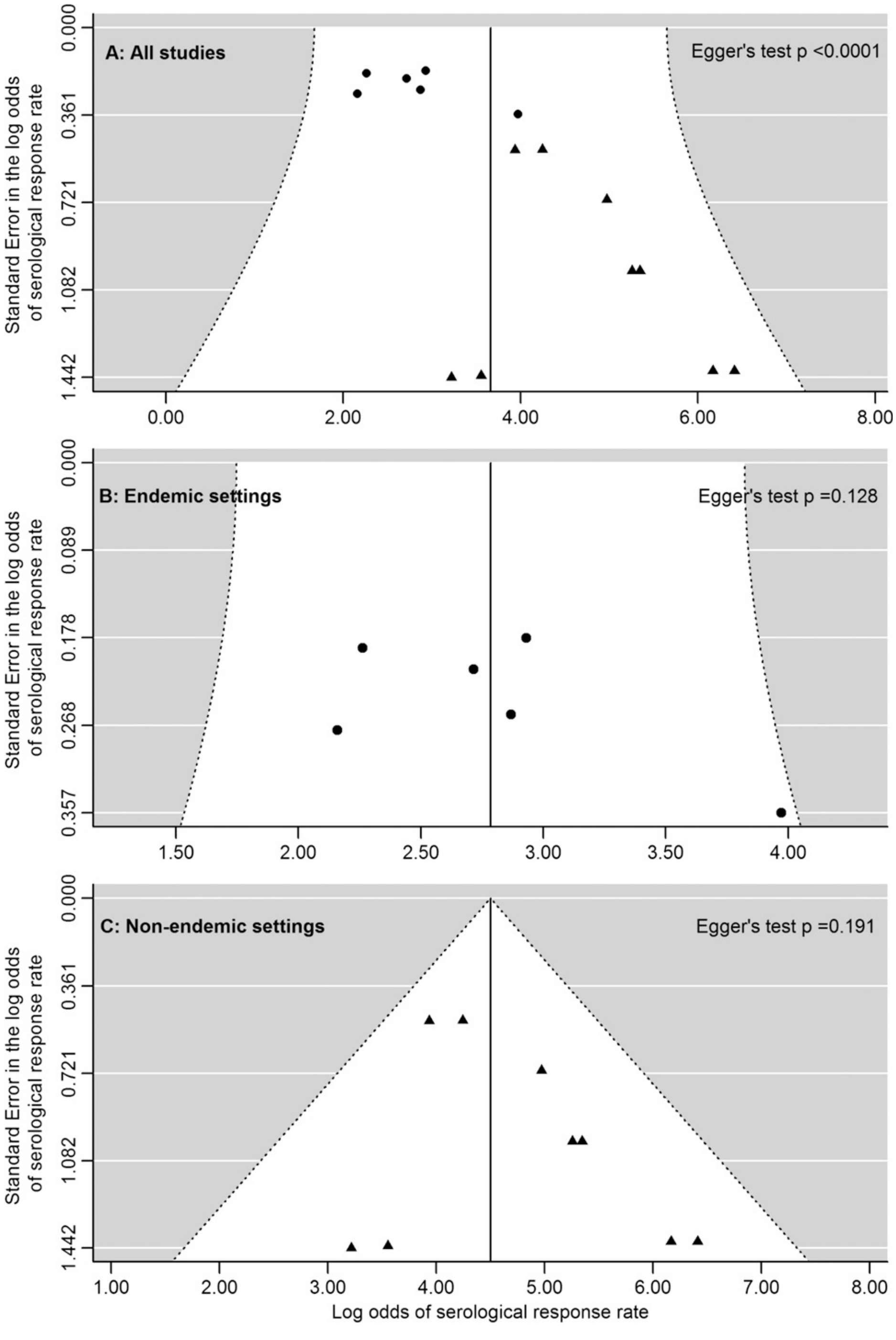

Supplemental Figure 1. Funnel plot, accounting for between-study heterogeneity. (**A**) All studies, (**B**) studies conducted in endemic settings or settings at transitional risk, and (**C**) studies conducted in nonendemic settings. Dots represent studies conducted in endemic settings or settings at transitional risk and triangles represent studies conducted in nonendemic settings.
